# Quality of life, morbidity, mortality, and long-term prognosis after craniopharyngioma

**DOI:** 10.3389/fendo.2026.1768254

**Published:** 2026-06-05

**Authors:** Hermann L. Müller

**Affiliations:** Department of Pediatrics and Pediatric Hematology/Oncology, University Children’s Hospital, Carl von Ossietzky Universität, Klinikum Oldenburg AöR, Oldenburg, Germany

**Keywords:** craniopharyngioma, hypothalamus, obesity, quality of life, semaglutide, setmelanotide

## Abstract

During the first months following diagnosis and treatment of childhood-onset craniopharyngioma, a substantial proportion of patients experience rapid and marked weight gain. This frequently progresses to severe hypothalamic obesity, which results from hypothalamic injury caused either by the tumor itself or by therapeutic interventions. Hypothalamic obesity should be regarded as one manifestation within the broader clinical spectrum of hypothalamic syndrome. Given the pivotal role of hypothalamic nuclei in maintaining physiological homeostasis, hypothalamic syndrome encompasses a wide range of disturbances, including hypothalamic–pituitary hormone deficiencies, disruption of circadian rhythms, impaired regulation of hunger, satiety, and thirst, as well as thermoregulatory dysfunction and cognitive, sleep-related, and psychosocial impairments. Consequently, affected individuals often develop persistent obesity, chronic fatigue, excessive daytime sleepiness, and mood disturbances, which may contribute to social withdrawal, academic challenges, reduced participation in daily activities, and impaired quality of life. Over time, these patients are at increased risk for metabolic syndrome, cardiovascular disease, sustained reductions in quality of life, and premature mortality. Historically, the management of hypothalamic syndrome has been challenging for both patients and clinicians, as conventional obesity treatments, including lifestyle modification, dietary interventions, and physical activity, have shown limited long-term efficacy. Pharmacological approaches have likewise been unsatisfactory, either due to insufficient effectiveness or unacceptable adverse effects leading to their withdrawal from clinical use. The therapeutic impact of central nervous system stimulants and glucagon-like peptide-1 receptor agonists in acquired hypothalamic obesity remains inconsistent and subject to ongoing debate. Recent findings from a randomized controlled trial provide, for the first time, encouraging evidence that setmelanotide, a melanocortin-4 receptor agonist, may substantially improve outcomes in patients with hypothalamic dysfunction associated with hypothalamic obesity. The emergence of a safe and effective pharmacological therapy that addresses not only metabolic abnormalities but also key psychosocial features, such as hyperphagia and overall quality of life, may represent a significant advancement in the management of this complex condition and offers the potential to meaningfully improve outcomes in this highly burdened and underserved patient population.

## 1 Introduction

Craniopharyngiomas (CPs) are rare, histologically benign tumors arising in the sellar and parasellar regions (1). According to the 2021 World Health Organization (WHO) classification of tumors of the central nervous system, adamantinomatous CP and papillary CP are recognized as two distinct pathological entities (2). Adamantinomatous CP predominantly occurs in pediatric populations (<18 years), with a median age at diagnosis between 5 and 9 years, whereas papillary CP almost exclusively affects adults, most commonly between 55 and 69 years of age. The adamantinomatous type is more common in both age groups, but papillary CP occur almost exclusively in adults The annual incidence of CP is estimated to range from 0.5 to 2.0 cases per million individuals (1). Although short-term overall survival rates are high, many survivors experience substantial impairments in health-related quality of life (QoL), largely attributable to hypothalamic dysfunction resulting from the tumor and/or its treatment (1, 3–7). This review presents an update of previous publications (4–7) on outcome after CP with special focus on QoL and future perspectives on improved management of weight development and psychosocial situation by novel glucagon-like peptide-1 receptor agonist (GLP-1RA) (semaglutide), and melanocortin-4 receptor (MC4R) agonist (setmelanotide) therapies.

## 2 Survival and late mortality

Overall mortality among patients with CP has been reported to be three- to fivefold higher than that observed in the general population (8). In pediatric cohorts, five-year survival rates range from 83% to 96% (9), ten-year survival from 65% to 100% (10), and long-term survival averages approximately 62% after 20 years of follow-up. While certain studies suggest more favorable long-term survival in younger patients, others report improved outcomes in older individuals (11, 12). The influence of sex on prognosis remains controversial, with some investigations indicating higher mortality (8), weight gain (13), and reduced QoL (14) among female patients whereas others report no significant sex-related differences (15, 16). Late mortality is predominantly associated with tumor- and/or treatment-related sequelae (17), including disease progression or recurrence, chronic hypothalamic dysfunction, endocrine insufficiencies, seizures, cerebrovascular complications (18, 19), and metabolic disorders such as nonalcoholic fatty liver disease (20, 21), which may progress to liver cirrhosis in some cases (10). A recent systematic review emphasized the substantial long-term morbidity associated with CP, reporting standardized mortality ratios ranging from 2.88 to 9.28. Cardiovascular mortality was found to be three- to nineteenfold higher compared with the general population, with the greatest risk observed in female patients (8, 22). The prognostic relevance of histological subtype remains a subject of debate. Some studies describe superior five-year survival in patients with papillary CP compared with those with adamantinomatous or mixed histology. In childhood-onset CP, better baseline functional status has been associated with improved ten-year survival, whereas the impact of hydrocephalus at diagnosis remains uncertain (15, 16, 23). Notably, Sterkenburg et al. (24) demonstrated significantly reduced 20-year survival in pediatric CP patients with hypothalamic involvement. In contrast, the extent of surgical resection did not significantly influence long-term progression-free survival, supporting the notion that radical gross-total resection does not confer a survival advantage in preventing recurrence.

Beckhaus et al. (11) analyzed the largest cohort of childhood-onset CP reported to date (n=709), focusing on clinical characteristics and long-term outcomes. At final follow-up, severe obesity (body mass index (BMI) >3SDS) was present in 45.4% of patients. Posterior hypothalamic involvement and hypothalamic lesions were identified as independent predictors of both reduced event-free survival and obesity. Overall survival was not associated with age at diagnosis; however, younger age (<12 years) emerged as a risk factor for disease progression and relapse. Children diagnosed before the age of 6 years exhibited lower event-free survival but reported higher QoL compared with those diagnosed at ≥6 years. Reduced functional capacity percentiles were associated with higher BMI SDS at last follow-up and with CP diagnosis before the age of 2 years. These findings suggest that differences in treatment strategies, such as the timing and modality of radiotherapy, or intrinsic tumor biology may contribute to outcome variability. Nevertheless, comparable overall survival across age groups indicates that (re-)irradiation and surgical reintervention can effectively control relapse or progression. Despite similar survival outcomes, long-term functional capacity varied considerably. Patients diagnosed at a younger age demonstrated poorer functional capacity during follow-up. Interestingly, both self-reported and parent-reported QoL assessments, were less favorable among patients diagnosed at an older age, particularly with respect to body image. Developmental factors may partly explain this discrepancy: while neurological and endocrine deficits in very young children profoundly affect overall development, older children and adolescents may be more aware of changes in QoL due to their ability to compare pre- and post-diagnosis functioning. Consequently, body image concerns may be more salient in adolescents, for whom physical appearance represents a central component of QoL (25).

## 3 Quality of life, neurocognitive outcome, and psychosocial functioning

In pediatric patients with CP, QoL is influenced by both tumor-related factors and treatment effects. Studies examining psychosocial and physical functioning report heterogeneous outcomes, with most patients demonstrating satisfactory functioning, while up to half experience significant impairments (10, 26, 27) (**Table 1**). Social and emotional domains are most frequently affected, with patients reporting lower psychosocial well-being compared with physical health (10). Additional difficulties include somatic complaints such as pain, reduced mobility, and limitations in self-care (10, 56). Behavioral assessments reveal elevated rates of psychopathological symptoms, including anxiety, depression, and social withdrawal (57). Common challenges in daily life include learning difficulties, emotional dysregulation, impaired peer relationships, and concerns regarding body image (58, 59). Factors associated with poorer QoL and adverse psychosocial and neurocognitive outcomes include younger age at diagnosis, preoperative functional impairment, and tumor characteristics such as large tumor volume or involvement of the hypothalamus and third ventricle. Treatment-related factors also play a role, with less favorable outcomes reported following radical surgery alone compared with limited surgical resection combined with radiotherapy, as well as in patients requiring multiple interventions for recurrence. Endocrine, neurological, and ophthalmological sequelae further compromise QoL (10, 26, 56, 60). Among these, hypothalamic dysfunction has been identified as the most critical determinant adversely affecting social functioning, physical capacity, and body image (9, 10, 61, 62).

**Table 1:** Health-related quality of life after craniopharyngioma. Selected publications (2019–2026).

Diagnosis	Pat. no.	Age at cranio-pharyngioma diagnosis (years)	Follow-up (FUP) interval (years)	Treatment	Quality of life/outcome	Authors/year of publication
CO CP grade 2 HI pre OP	109	Median 9.5 (range: 1.3 – 17.9)	Mean 6.1 (3.0 – 10.2)	23 grade 0 HL, 29 gra-de 1 HL, 57 grade 2 HL post OP	Worse PEDQOL for grade 3 patients in terms of physical, social and emotional functionality when compared with HL grade 0 and 1.	Bogusz et al., 2019 (28)
CO CP	131	Median 9.7 (range: 1.3 – 17.6)	3 years	21 (18%) CR; 94 (82%) IR	Grade 2 HI, grade 2 HL post OP and CR were associated with low QoL.	Eveslage et al., 2019 (29)
CO CP + HL	290	n.a.	n. a.	n. a.	Worldwide online survey: Obesity (51%) and fatigue (48%). Needs for improvement in the domains of obesity, fatigue and lifestyle.	Van Roessel et al., 2020 (30)
CO CP	78	Mean 10.8 ± 3.11 SD	n. a.	56 surgical resections 16 catheter implant	Poorer parental-reported QoL; AVP-D directly predicted greater global executive functioning impairment.	Niel et al., 2021 (31)
Caregivers of CO CP	106	<18	n. a.	48 RT; 134 surgical interventions	Online survey: reduced social functioning.	Craven et al., 2022 (32)
CO CP, pare-nts/caregivers	120	Median 10.0 (range: 1.3 –16.8)	3	25 CR, 95 IR; 61 RT	Reduced autonomy 3 years after diagnosis in self-assessments and parental assessments of QoL (PedQol).	Sowithayasakul et al., 2023 (33)
CO CP with RT	99	Median 9.5 (range: 1.6 – 17.9)	Median 6.4 (0.9 – 14.7)	64 PBT 35 photon-based RT	No significant difference between PBT and photon-based RT in terms of QoL (PedQol), functional capacity (FMH), and BMI.	Friedrich et al., 2023 (34)
CO CP	709	< 2 years: 3% 2-5: 16%; 6-11: 46%; 12-18: 35%	Median 8.37 (0.04 – 38.87)	33% RT: 35% PBT; 46% photon-based RT; 27% CR	BMI>3SD in 45%; risk factor for obesity: HI and HL. Patients <2years at diagnosis: low functional capacity (FMH); Patients >12 years at diagno-sis: low QoL (PedQol); Lower event-free survival in younger age groups.	Beckhaus et al., 2023 (11)
Caregivers of CO CP	82	Mean 9.3 ± 4.5 SD	Duration care-giving: median: 5.5 (<1 – 28)	52.4% CR	Survivor poly-symptomatology predicted caregiver burden. The study separated hyperphagia and obesity and identified hyperphagia and other hypothalamic dysfunction symptoms as understudied issues.	Kayadjanian et al., 2023 (35)
CO CP	87	Mean 7.39 ± 3.67 SD	Median 6.54 (IQR: 3.1–10.7)	25% CR; 44% IR; 30% cyst drain; 46% RT	BMI at diagnosis and grade of HL were associated with HO	Van Schaik et al., 2023 (36)
CO CP	48	CR: Median 6.4 (2.2–16.8) IR+RT: 8.5 (3.8 – 16.4)	10	21 CR, 22 IR+RT	No differences in the trajectory of intellectual functioning or QoL scale scores between the groups (CR vs. IR+RT).	Aldave et al., 2023 (37)
AO CP: 90% CO CP: 10%	109	Median 40.0 (range: 28.5 – 56.0)	Median 10.0 (2.5 – 24.0)	25.6%: surgery 4.6%: RT	SF-36: impaired QoL compared with general population. MCS, GAD7, PHQ9: adverse effect of AVP-D in multi-variate linear regression. AVP-D risk factor for developing depressive symptoms.	Lin B et al., 2024 (38)
CO CP from LMIC	29	Mean 13.5 ± 4.2 SD	Mean: 4.4 ± 2.2 SD	15 CR; 11 debulking, 3 reservoir/biopsy	PedsQL: CR 56.6 ± 7.12, Debulking: 93.8 ± 3.37; Biopsy: 83.3 ± 5.69; lowest QoL score after CR.	Baqai et al., 2024 (39)
CO CP	11	Median 15.2 (IQR: 9.7 – 17.9)	Mean 5.3 ± 3.2 (SD)	73% surgery 82% RT	PEDS-QL4.0: Worse QoL in global, physical, emotional, and psycho-social dimensions linked to HI. Worse global QoL after RT.	Pereira Neto et al., 2024 (40)
CO OP	92	Mean 10.5 ± 4.0 SD	n. a.	PBT after surgical intervention	Fatigue, QoL, and brain tumor symptoms improved over time during PBT.	Mandrell et al., 2024 (41)
CO CP	66	Median 5 (IQR: 3 – 8)	Median 7.4 (IQR: 2.8 –9.7).	100% surgery; 44% RT; 24% cystic therapy	PedsQL: QoL was impaired by repeated surgeries, RT, and longer FUP interval.	Perez-Torres et al., 2024 (42)
CO CP	108	Median 7 (IQR: 4.9 – 11.2)	Median 9.7 (IQR: 5.9–13.6)	90% hypothalamus-spa-ring surgery, 74% RT	10.2% of patients have regular psychiatric or psychological follow-up	Rovani et al., 2024 (13)
CO CP	119	Median 12 (range: 2 – 17)	Mean 10 (1 – 39)	29% CR; 6 grade 0, 23 grade 1, 55 grade 2 HL	QoL (EORTC QLQ-C30) negatively correlated with daytime sleepiness (ESS), highest ESS in HL grade 2.	Mann-Markutzyk et al., 2025 (43)
CO CP with PBT	47	Median 9.7 (range: 2.4 – 22.1)	Median 11.2 (1.3 – 17.3)	55% surgery 100% RT (PBT)	PedsQL: PPR and CSR TCS lower than normal controls at last FUP, and lower in patients with AVP-D, sex hormone deficiency, and hyperphagia	Rose et al., 2025 (44)
AO CP (n=1), CO (n=3); HL	4	6, 7, 8, 49	FUP: range 16 – 49	Semaglutide 1.7-2.4 mg/wk, 24 months	Interviews analyzed by inductive approach (45): improved QoL (physical, social function, work life)	Gjersdal et al., 2025 (46)
AO CP	26	n. a.	n. a.	100% surgery	New 39-item Craniopharyngioma QoL instrument showed reduced and different ratings for patients and providers	Myneni et al., 2025 (47)
AO CP	1	21	4 months tirze-patide therapy	GLP1R agonist - tirzepatide	Weight loss 9 kg over 4 months; decrease in BMI (initial: 40 kg/m^2^, after 4 mo: 37 kg/m^2^). Rebound weight gain after end of tirzepatide therapy	Brijmohan et al, 2025 (48)
AO CP (n=26) CO CP (n=14)	40	28 (0 – 75)	80 (3 – 229) months	Retrospective analysis of social participation	Larger tumor size and recurrence significantly deteriorate the rate of patients’ social participation during long-term FUP	Umeda et al., 2025 (49)
AO CP (n=16), CO CP (n=5)	21	median 24.0 (IQR: 17.0 – 54.0).	123 months (IQR 36–226)	95% surgery, 30% RT	Modified PWS Behavior Questionnaire (PWSBQ): similar impairments in AO and CO	Tavares de Silva et al., 2026 (50)
Caregivers of CO CP	40	HO: 7.9 (4.1 SD); non-HO: 9.9 (13.2)	HO: 9.8 (6.5); nonHO:3.3(4.1)	43% HO, 57% non-HO	QoL impaired due to HL associated with low satiety, higher hunger/food preoccupation	Kayadjanian et al., 2026 (51)
AO CP	25	44 (range: 22–55)	Age at study: 49 (36–59)	84% surgery 80% RT	88% reported low mood, 68% felt they no longer recognized themselves, 56% missed social events due to anxiety	Daughters et al., 2026 (52)
AO CP	32	46.7 ± 22.9	12 months	Endoscopic endonasal resection of AO CP	no decline in Sinonasal Outcome Test (SNOT-22) and Anterior Skull Base Questionnaire (ASBQ-35) 12 months after surgery	Banting et al., 2026 (53)
AO (n=476) CO (n=128)	604	2 – 76	n. a.	Endoscopic endonasal approach (90% CR)	EORTC QLQ-C30: Poorer QoL for physical function, role function, and cognitive function in CP with HL compared to CP without HL. Higher global QoL scores in sellar/suprasellar CP without HL than in CP with HL and 3. ventricle involvement.	Cai et al., 2026 (54)
HO, CO CP (n=93)	120	81 Setmelanotide: 19.2 ± 13.0 (range 4–65); 39 Placebo: 21.4 ± 15.5 (4–66)	n. a.	Phase 3 RCT (2:1): 81 setmelanotide (1.5–3.0 mg/d), 39 placebo; 52 weeks	BMI change: Setmelanotide -16.5%, placebo: +3%; QoL assessed by IWQOL-Lite-CT, IWQOL-Lite-CT physical functioning score, IWQOL-Kids total score; IWQOL caregiver-reported proxy showed significantly improved QoL in the setmelanotide group	Miller et al., 2026 (55),

AO CP, adult-onset craniopharyngioma; AVP-D, arginine vasopressin deficiency; CO CP, childhood-onset craniopharyngioma; CR, complete resection; CSR, Child-self report; ESS, Epworth Sleepiness Scale; FMH, functional ability scale Münster Heidelberg; FUP, follow-up; GAD7, Generalized Anxiety Disorder Questionnaire scale; QoL, quality of life; HI, presurgical hypothalamic involvement; HL, surgical hypothalamic lesions; HO, hypothalamic obesity; IQR, interquartile ratio; IR, incomplete resection; IWQOL-Lite-CT, Impact of Weight on Quality of Life-Lite Clinical Trials version; LMIC, lower-middle-income country; n. a., data not available; PBT, proton beam therapy; PEDS-QL4.0., Pediatric Quality of Life Inventory; PHQ9, Patient Health Questionnaire Depression; PPR, parent-proxy reports; RT, radiotherapy; RCT, randomized controlled trial; SD, standard deviation; SF-36, Short Form 36; TCS, total core scores.

Modified from Müller HL, 2025 (7) with permission of mdpi.

Long-term neurocognitive sequelae following treatment of childhood-onset CP primarily involve deficits in attention, executive functioning, episodic memory, and working memory (10, 58, 59, 63). Özyurt et al. (64, 65) demonstrated that hypothalamic damage alters neural correlates of memory retrieval within the medial prefrontal cortex, reflecting reduced efficiency of executive control networks, likely mediated by disrupted thalamic connectivity. Subtotal resection followed by radiotherapy has also been associated with persistent psychological and educational impairments (58), including memory disturbances, slower cognitive processing, attention deficits, and behavioral instability (58, 59, 63). Although overall intellectual functioning remains preserved in up to 82% of patients, deficits in visual memory have been observed despite intact visuospatial abilities (58, 59). These higher-order cognitive impairments, particularly attentional deficits, are considered major contributors to academic underachievement.

Despite extensive documentation of neurocognitive impairments, evidence on targeted interventions remains scarce. Case reports have described cognitive rehabilitation approaches, such as goal management training combined with environmental modifications, which resulted in measurable improvements in tasks requiring structured behavior (66). Behavioral interventions utilizing functional behavioral analysis and differential reinforcement have been shown to reduce maladaptive behaviors while promoting adaptive coping strategies. Collectively, these findings suggest that cognitive and behavioral rehabilitation may help survivors compensate for persistent cognitive and psychosocial deficits.

## 4 Visual impairment

Van Schaik et al. reported that 42% of patients with childhood-onset CP presented with visual field defects at diagnosis. Reduced visual acuity was observed unilaterally in 19% of patients and bilaterally in eight individuals (19%) (36). Data from the German KRANIOPHARYNGEOM Registry 2019 indicated that 70% of patients exhibited some degree of visual impairment at diagnosis. At three months after diagnosis, visual deficits persisted in 51% of affected patients, and at three-year follow-up, 54% continued to experience visual impairment (33). Although visual deficits affected patient autonomy and family functioning, overall QoL scores did not differ significantly between patients with and without visual impairment (33).

## 5 Hypopituitarism

The hypothalamic nuclei regulate pituitary function through secretion of releasing hormones, including corticotropin-releasing hormone, growth hormone (GH)-releasing hormone, gonadotropin-releasing hormone, and thyrotropin-releasing hormone. Consequently, damage to hypothalamic structures may result in multiple pituitary hormone deficiencies, such as hypogonadotropic hypogonadism, GH deficiency, central hypothyroidism, and adrenocorticotropic hormone deficiency (67). In addition, the hypothalamus synthesizes arginine vasopressin (AVP), which is stored and released by the posterior pituitary. Patients with hypothalamic dysfunction may therefore develop AVP deficiency (AVP-D), formerly termed central diabetes insipidus, or less commonly the syndrome of inappropriate antidiuretic hormone secretion (SIADH). Following surgical treatment for CP, approximately 80–90% of patients develop panhypopituitarism (16). The extent of endocrine dysfunction is influenced by surgical technique, with transcranial approaches associated with a higher risk of pituitary deficits than transsphenoidal surgery (53, 54). Furthermore, gross total resection carries a greater likelihood of endocrine sequelae than subtotal resection, particularly when combined with adjuvant radiotherapy (1, 11).

### 5.1 Growth hormone deficiency

Insufficient GH replacement is associated with significant adverse outcomes, including reduced muscle mass, diminished exercise tolerance, increased abdominal adiposity, elevated cardiovascular mortality risk (8), low energy expenditure, muscle weakness, impaired QoL, and growth failure (24). Initiation of GH replacement during childhood has been shown to improve linear growth without adversely affecting overall survival or progression-free survival (68). Early treatment in pediatric patients is also associated with sustained long-term improvements in QoL (69). Importantly, GH replacement in patients with confirmed deficiency does not increase the risk of CP recurrence or progression (70). Unlike recommendations for many malignant tumors, no mandatory waiting period after completion of oncological therapy is required before commencing GH therapy in CP patients. Early initiation following diagnosis may also contribute to improved weight regulation and QoL (69). Adults with childhood-onset CP have a markedly increased cardiovascular mortality risk, estimated at 3- to 19-fold higher than the general population (8). GH replacement may reduce cardiovascular risk while improving body composition and bone mineral density. A recent consensus statement from the Growth Hormone Research Society concluded that GH replacement therapy is safe in individuals with CP (70, 71). Although GH therapy does not directly resolve hypothalamic obesity, it improves body composition, mitigates metabolic complications, and enhances QoL in CP survivors (69).

### 5.2 Adrenocorticotropin deficiency

Adrenocorticotropin (ACTH) deficiency, commonly resulting from tumor-related damage or treatment interventions in CP, requires hydrocortisone replacement with dose adjustments during physiological stress. Cortisol deficiency may present with headache, nausea, and reduced stress tolerance, and untreated deficiency may progress to life-threatening adrenal crises. Children with acquired hypothalamic dysfunction may exhibit increased 11β-hydroxysteroid dehydrogenase type 1 activity, suggesting that lower hydrocortisone replacement doses (6–8 mg/m²) may be appropriate compared with children without hypothalamic involvement. Excessive or non-physiological glucocorticoid replacement is associated with increased obesity risk. Although perioperative cortisone administration may contribute to short-term weight gain, it has not been linked to persistent obesity. When prescribed in physiological doses, hydrocortisone should not substantially increase the risk of weight gain (72). Individualized dose optimization is therefore essential to maintain metabolic stability while preventing adrenal crises and hypoglycemia. Comprehensive education of patients and families is equally important to enable recognition of stress-related situations and timely emergency management.

### 5.3 Hypothyroidism

Central hypothyroidism may remain unrecognized when free thyroxine (T4) concentrations remain within the lower portion of the reference range. A decline of more than 20% in free T4 over time should prompt suspicion and consideration of levothyroxine replacement (73). Therapy should be titrated to maintain serum free T4 concentrations within the mid-to-upper normal range. Inadequate replacement may contribute to weight gain, emphasizing the importance of careful monitoring and dose adjustment.

### 5.4 Gonadotropin deficiency

Gonadotropin deficiency frequently necessitates induction of puberty and in selected cases medically assisted reproduction (74). Adults treated for CP during childhood exhibit higher rates of psychosexual dysfunction and reduced sexual activity (74). In males, hypogonadism with low testosterone concentrations is associated with an adverse metabolic profile and increased mortality risk. Timely and adequate testosterone replacement can improve body composition by reducing fat mass and increasing lean muscle mass. Infertility and reduced bone mineral density may affect individuals of both sexes, while men may additionally develop increased adiposity and anemia (67, 75).

### 5.5 Arginine vasopressin deficiency

AVP-D commonly occurs following hypothalamic injury, particularly when posterior hypothalamic regions are affected (76). It may therefore serve both as a clinical marker of hypothalamic damage and as an independent risk factor for hypothalamic obesity (77) and impaired QoL (38). When hypothalamic thirst regulation is also disrupted, patients may develop adipsic AVP-D (78) or adipsic SIADH (79). These conditions carry substantial risk for severe sodium imbalance, impaired homeostasis, and recurrent emergency hospitalizations. AVP-D management requires close monitoring of body weight, fluid intake, and urine output, often combined with strict fluid scheduling and individualized desmopressin dose adjustment. Increased risks of sodium disturbances and heart failure contribute significantly to morbidity and mortality in CP patients with AVP-D (78).

### 5.6 Oxytocin

Oxytocin deficiency in CP primarily results from hypothalamic injury caused by the tumor itself or its treatment and may contribute to development of hypothalamic syndrome including hypothalamic obesity (80, 81). Compared with healthy controls, CP patients demonstrate lower basal and stimulated salivary oxytocin concentrations (82–85). Reduced oxytocin levels have been associated with increased anxiety and impaired social cognition. Patients with anterior hypothalamic lesions appear to have particularly low salivary oxytocin concentrations and may show the most favorable neuropsychological response to a single dose of intranasal oxytocin compared with patients with posterior hypothalamic lesions or healthy controls (83, 84). Converging evidence from two independent studies indicates an association between changes in salivary oxytocin concentrations before and after stimulation and both BMI and eating behaviors in patients with CP. Specifically, Daubenbüchel et al. (83) reported that higher BMI in affected patients was associated with a smaller reduction in salivary oxytocin concentrations from the pre-prandial to the post-prandial state, whereas no such relationship was observed in healthy controls (86). These observations suggest that oxytocin-based therapies may represent a promising future treatment strategy for hypothalamic syndrome and hypothalamic obesity in CP (86–88).

## 6 Hypothalamic syndrome

Hypothalamic syndrome is an umbrella term describing a constellation of clinical manifestations resulting from hypothalamic damage or dysfunction (5–7, 89–91). Sterkenburg et al. demonstrated that hypothalamic involvement in CP is associated with reduced overall survival (24). While hypothalamic syndrome is frequently observed in CP (92), it also occurs in other conditions, including low-grade glioma (93), germ cell tumors (94), traumatic brain injury (95, 96), and Prader–Willi syndrome (97). Importantly, hypothalamic syndrome is distinct from hypothalamic obesity, as it encompasses a broader range of symptoms that vary considerably between individuals (98, 99). According to van Santen et al., diagnosis should be based on five core clinical domains: disordered eating behavior, behavioral disturbances, sleep disorders, impaired temperature regulation, and endocrine deficiencies (100). Recently, an update of the diagnostic score for hypothalamic syndrome has been published, focusing on the domain of autonomic dysregulation (90, 101). The combined burden of these domains leads to a profound reduction in health-related QoL for affected patients (102) along with high costs of health care utilization (103).

### 6.1 Obesity and eating disorders

Under physiological conditions, body weight and appetite regulation are maintained through a balance between orexigenic signals, such as neuropeptide Y and ghrelin, and anorexigenic signals, including insulin and leptin (104). Damage to key hypothalamic structures, particularly the arcuate and ventromedial nuclei, as well as the nucleus tractus solitarius disrupts these regulatory pathways (104, 105). This disruption promotes insulin and leptin resistance, leading to impaired control of hunger and satiety and manifesting clinically as disordered eating behavior (106). Patients with hypothalamic obesity frequently exhibit dominance of the parasympathetic nervous system due to increased vagal activity (101). Concurrently, reduced sympathetic tone lowers total energy expenditure, thereby favoring hyperinsulinemia and excessive fat accumulation (106). With regard to imaging, hypothalamic T2 signal intensity (SI), suggestive of inflammation or edema, rises after surgery. Yurddas et al. (107) observed that high hypothalamic post-operative T2 SI correlated with BMI increase and presence of post-surgical hypothalamic syndrome. High hypothalamic post-operative T2 SI was linked to older age, less cystic tumors, and higher Müller grade of hypothalamic damage.

Longitudinal studies indicate that approximately 40–60% of long-term CP survivors with hypothalamic involvement develop hypothalamic obesity (9, 82, 108, 109). In a French cohort comprising 108 patients with CP, 54% were classified as overweight or obese. Female sex, hypothalamic involvement, and a baseline BMI exceeding 2 SDS were identified as significant risk factors for progressive obesity (13). Furthermore, Beckhaus et al. demonstrated that familial predisposition to obesity, reflected by parental BMI at the time of CP diagnosis, is associated with an increased risk of severe hypothalamic obesity in affected patients (110).

A recently published consensus on the diagnosis of acquired hypothalamic obesity proposed the following diagnostic criteria (111):

• the presence of a traumatic event or (oncological) disease resulting in hypothalamic lesions detectable by magnetic resonance imaging (MRI),
• a rapid (within the first 12 months after surgery or diagnosis), persistent (lasting at least 24 months), and clinically relevant increase in BMI (≥5% BMI increase in adults or ≥1.0 SDS BMI increase in pediatric patients), documented under clinical and anthropometric monitoring at three-month intervals and beginning within the first year following hypothalamic injury,
• the presence of obesity above defined thresholds (BMI SDS ≥+2.0 in pediatric patients; BMI ≥25 kg/m² or ≥30 kg/m² in adults, adjusted for racial and ethnic characteristics).

Once severe obesity is established, meaningful weight reduction is rarely achievable (106), and BMI typically stabilizes at an elevated plateau (24). To date, no standardized treatment strategy exists for this multifactorial condition. Current management approaches emphasize prevention of hypothalamic damage through hypothalamus-sparing surgical techniques, the use of advanced radiotherapy modalities such as proton beam therapy, and pharmacological interventions specifically targeting hypothalamic obesity (96, 112–114).

### 6.2 Circadian rhythms, fatigue, and daytime sleepiness

Sleep-related disturbances are a frequent manifestation of hypothalamic syndrome in patients with CP (115). Commonly reported disorders include insomnia, secondary narcolepsy, excessive daytime sleepiness, fragmented sleep, circadian rhythm sleep-wake disorders, and sleep-related breathing disorders such as obstructive sleep apnea (116). Hypersomnia is defined as a clinically significant degree of daytime sleepiness that occurs despite normal nocturnal sleep duration and quality, often resulting in difficulty maintaining wakefulness and unintended sleep episodes during daily activities (116). Müller et al. evaluated 115 individuals with childhood-onset CP and identified 35 patients with excessive daytime sleepiness, including four cases consistent with secondary narcolepsy and three cases of hypersomnia (117). Treatment with central nervous system stimulants, such as modafinil and methylphenidate, resulted in improved alertness and daily functioning in these patients (117). In another cohort of 70 childhood-onset CP patients, hypersomnia was diagnosed in 41% (n=29) following surgery but prior to radiotherapy (118). Sterkenburg et al. reported increased physical fatigue and reduced motivation among CP survivors with hypothalamic involvement (24). Tumor-related neurological deficits may further contribute to daytime sleepiness, insomnia, and circadian rhythm disturbances (116). Fatigue represents a major determinant of reduced QoL in CP survivors (119).

### 6.3 Physical activity and energy expenditure

Accelerometry-based assessments have demonstrated that patients with CP exhibit significantly lower levels of physical activity compared with BMI-matched healthy controls (120). Severe hypothalamic obesity in CP is frequently accompanied by excessive daytime sleepiness (121). Additionally, affected patients often display reduced early morning and nocturnal salivary melatonin concentrations, which correlate with both hypothalamic obesity and daytime sleepiness. Melatonin supplementation has been shown to normalize salivary melatonin levels and induce short-term improvements in physical activity and reductions in daytime sleepiness (122). However, data on the long-term efficacy of melatonin treatment with respect to sleepiness and hypothalamic obesity are currently lacking.

Polysomnographic studies in CP patients with hypothalamic obesity and excessive daytime sleepiness reveal features characteristic of hypersomnia and secondary narcolepsy, including frequent sleep-onset rapid eye movement (SOREM) periods. Pharmacological treatment with stimulants such as methylphenidate and modafinil has been shown to substantially alleviate daytime sleepiness in these patients (117). Moreover, hypothalamic obesity and weight gain in CP are associated with metabolic alterations, including reduced resting energy expenditure (REE) (123–126).

### 6.4 Psychosocial skills

Accurate perception and interpretation of others’ mental states are fundamental for effective social interaction. Deficits in these psychosocial skills impose considerable challenges not only for patients but also for their families and social environments, adversely affecting academic performance and occupational functioning. Özyurt et al. demonstrated that CP patients with hypothalamic lesions exhibit reduced ability to understand others’ thoughts and to accurately interpret social cues, intentions, and emotions compared with healthy controls. These impairments are commonly associated with dysfunction in brain regions critical for social cognition, including limbic, frontal, and temporal structures. In CP, such deficits may result from tumor-related or treatment-induced brain injury and may exacerbate manifestations of hypothalamic syndrome (85).

## 7 Management

### 7.1 Management of hypothalamic syndrome

Van Iersel et al. proposed an individualized treatment algorithm for patients with CP (90, 101, 106). Following comprehensive diagnostic evaluation, therapeutic interventions may be implemented to address one or more of six key clinical domains: eating disorders, behavioral disturbances, sleep disorders, temperature dysregulation, and endocrine dysfunction.

In a systematic review on feasibility, safety, and efficacy of dietary or lifestyle interventions for hypothalamic obesity, van Roessel et al. (127) observed that the majority of available studies are limited by small sample sizes, lack of control groups, and a generally high risk of bias. Lifestyle interventions led to reductions in BMI primarily in the short-term, suggesting that sustained benefits likely require ongoing support and structured guidance. Overall, the evidence base regarding the feasibility and effectiveness of dietary and lifestyle interventions in hypothalamic obesity remains limited.

### 7.2 Pharmacological management of hypothalamic obesity

Pharmacological agents such as central stimulants (128–130), antidiabetic substances, including metformin (131), diazoxide, and tesofensine (132), used either as monotherapy or in combination, can improve insulin sensitivity and limit further weight gain (88, 133).

#### 7.2.1 Glucagon-like peptide-1 receptor agonist treatment

The introduction of GLP-1RA therapies has yielded substantial benefits for individuals with polygenic, multifactorial obesity (48, 134–136). However, evidence regarding their efficacy in disorders involving the MC4R pathway, such as acquired hypothalamic obesity, remains limited and inconsistent, and no GLP-1RA has been approved for hypothalamic obesity at the time of publication (137). Two randomized, placebo-controlled trials evaluating exenatide in acquired hypothalamic obesity did not demonstrate significant weight loss compared with placebo (138). A Phase 3 trial of once-weekly exenatide or placebo for 36 weeks in 42 participants with acquired hypothalamic obesity found a significant decrease in total body fat mass and waist circumference, but no significant difference in BMI change (139). A recent retrospective study of patients with hypothalamic obesity secondary to CP reported moderate weight reduction across various GLP-1RA therapies, although with marked interindividual variability over a mean follow-up period of 44 months (140). Additional retrospective analyses focusing on semaglutide have shown significant weight loss (141) and improvements in glycemic control and appetite regulation (142) in patients with hypothalamic obesity. The heterogeneity in treatment response may partly reflect submaximal dosing in this population (140).

#### 7.2.2 Melanocortin-4 receptor agonist treatment

The MC4R agonist setmelanotide has recently been approved by the U.S. Food and Drug Administration for the treatment of excess body weight and its long-term maintenance in both adult and pediatric patients aged ≥4 years with acquired hypothalamic obesity (15, 64 Platzhalter Packiungsbeilage IMCIVREE) (143) [US package insert]. Boston, MA: Rhythm Pharmaceuticals; 2024.). Preclinical evidence from a rat model of acquired hypothalamic obesity demonstrated that treatment with setmelanotide reduced food intake and resulted in significant weight loss (144). These findings are consistent with clinical observations from Phase 2 and Phase 3 trials, which have shown robust and clinically meaningful responses to setmelanotide in patients with acquired hypothalamic obesity (145) (55).

In a multicenter, open-label Phase 2 study (ClinicalTrials.gov identifier: NCT04725240), conducted across five sites in the United States, 18 patients with a history of hypothalamic injury or non-malignant hypothalamic tumors, treated with surgery, chemotherapy, or irradiation, received at least one dose of setmelanotide (145). After 16 weeks, 16 patients (89%) achieved the primary endpoint of ≥5% reduction in BMI from baseline (P<0.0001), with an overall mean BMI reduction of 15% ± 10%. Among participants aged ≥12 years, a 45% reduction in mean hunger scores from baseline was observed at week 16. The most frequently reported adverse events included nausea (61%), vomiting (33%), skin hyperpigmentation (33%), and diarrhea (22%) (145).

An international, randomized Phase 3 trial (ClinicalTrials.gov identifier: NCT05774756) further evaluated the efficacy and safety of setmelanotide in individuals with acquired hypothalamic obesity (55). Participants aged ≥4 years with hypothalamic obesity due to hypothalamic tumor, lesion, or injury were randomized to receive either setmelanotide (initiated at 0.5 mg once daily and titrated up to 1.5–3.0 mg/d depending on body weight, age, and tolerability) or placebo over a 52-week period. Eligibility criteria included a BMI ≥95^th^ percentile for participants aged 4 to <18 years or ≥30 kg/m² for adults. The study enrolled 120 participants (setmelanotide: n=81; placebo: n=39) with a mean age of 19.9 years. Treatment with setmelanotide resulted in a placebo-adjusted least-squares mean reduction in BMI of −19.8% (P<0.001). In participants aged ≥12 years, the reduction in weekly average maximal daily hunger score was significantly greater in the setmelanotide group compared with placebo (−2.73 *vs*. −1.45; P = 0.009). Furthermore, 80% of treated patients achieved at least a 5% BMI reduction at 52 weeks (146). Adverse events were generally mild to moderate in severity and were consistent with those observed in earlier trials. The most common included skin hyperpigmentation (55.6% *vs*. 7.7% in placebo), nausea (50.6% *vs*. 30.8%), vomiting (39.5% *vs*. 17.9%), and headache (38.3% *vs*. 30.8%) (55). Overall, the safety profile aligned with findings from the Phase 2 study (145) (55). Additional secondary outcome measures included the proportion of patients achieving a ≥2-point reduction in the weekly average maximal daily hunger score, as well as changes in the weekly average Symptoms of Hyperphagia composite score (147). In 81 evaluable patients aged ≥12 years, the least square mean change in the weekly average of the maximal daily hunger score among patients receiving setmelanotide was −2.73 (95% CI, −3.28 to −2.18) and in participants receiving placebo was −1.45 (95% CI, −2.23 to −0.67; P = 0.009). Accordingly, setmelanotide proved to be the first pharmacological agent with significant effect on pathological eating behavior such as hyperphagia.

QoL was evaluated in the phase 3 setmelanotide trial (55) using changes in the total score of the Impact of Weight on Quality of Life-Lite Clinical Trials version (IWQOL-Lite-CT), in the IWQOL-Lite-CT physical functioning subscale, and in the IWQOL-Kids total score for participants aged ≥11 to <18 years who were able to complete self-assessments (148). For participants within this age range who were unable to self-report, a caregiver-completed IWQOL parent-proxy questionnaire was administered. IWQOL-Lite-CT total score improved significantly under setmelanotide 32.0 (25.8 to 38.3) when compared with placebo 3.0 (−5.6 to 11.6). Most impressive improvement was detectable for the physical function domain.

A long-term extension study evaluating outcomes and QoL beyond one year of setmelanotide treatment is currently ongoing. Two Phase 2 clinical trials investigating other MC4R agonists, bivamelagon (ClinicalTrials.gov identifier: NCT06046443) and RM-718 (ClinicalTrials.gov identifier: NCT06239116), are underway in patients with CP and acquired hypothalamic obesity.

## 8 Prevention of hypothalamic dysfunction through hypothalamus-sparing approaches

**Figure 1:**
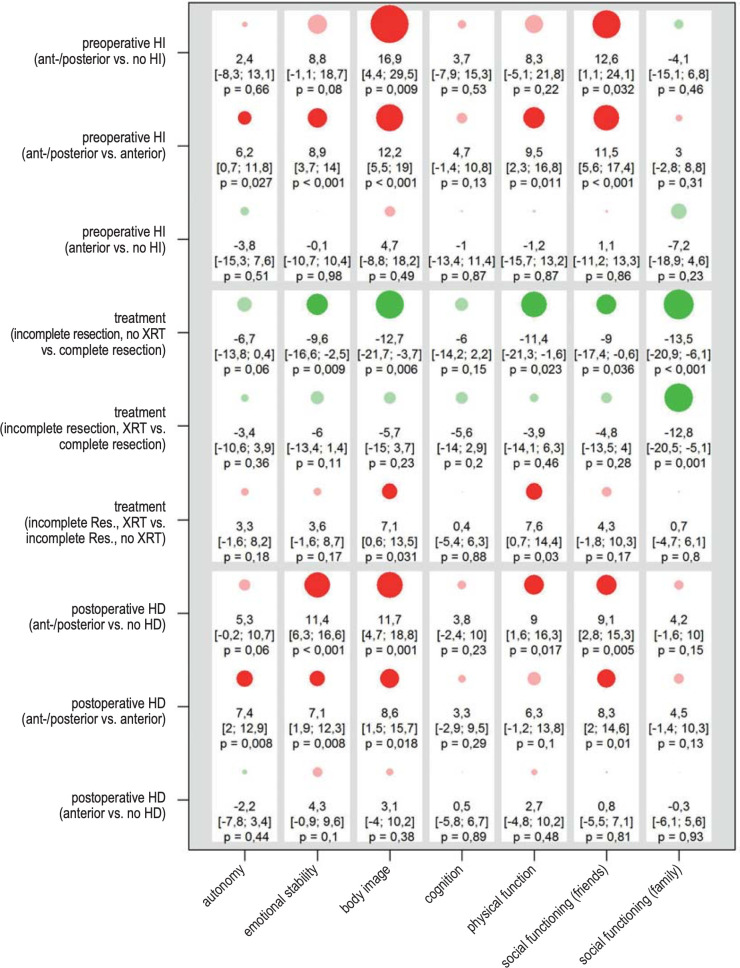
Results of the linear mixed models for the self-assessed quality of life (PEDQOL) in a cohort of 131 craniopharyngioma patients recruited in KRANIOPHARYNGEOM 2007 between 2007–2016. The PEDQOL score was modeled separately in each domain as a function of time, the factor in question, and the interaction between time and the factor in question. The results are displayed as differences of least-square estimates, with the associated 95%-confidence intervals and p values, describing the mean difference in PEDQOL scores between the two categories of each factor. The size of the circles is proportional to the mean difference; circles representing positive and negative effects on QoL are colored green and red, respectively (opaque when p ≤0.05). A box corresponds to the results of a model. The higher the PEDQOL score, the lower the subjective QoL; a minus sign thus indicates a beneficial influence on the QoL. (Reproduced from Eveslage et al., Dtsch Aerztebl Int 2019 (29), with kind permission of Deutsche Ärzteblatt International). HD, hypothalamic damage; HI, hypothalamic involvement, QoL, quality of life; PEDQOL, Pediatric Quality of Life Questionnaire; Res, resection; XRT, radiotherapy.

Eveslage et al. reported that both preoperative hypothalamic involvement and surgical injury affecting anterior and posterior hypothalamic regions are associated with reduced QoL in patients with CP. Furthermore, complete tumor resection was linked to poorer QoL outcomes compared with partial resection (**Figure 1**) (29). Preexisting hypothalamic involvement, particularly of posterior regions, constitutes a major risk factor for adverse outcomes in CP (7, 149–151). These risks may be mitigated through risk-adapted, hypothalamus-sparing surgical strategies designed to minimize additional hypothalamic damage (60, 152–154). Lesions confined to the anterior hypothalamus are generally associated with fewer long-term sequelae, whereas posterior hypothalamic damage is strongly linked to the development of hypothalamic syndrome and reduced QoL (28). In this context, radiotherapy represents an effective modality for controlling residual or recurrent disease and is an essential component of hypothalamus-sparing treatment strategies for CP (1).

## 9 Transition from pediatric to adult care

The principles of high-quality transitional care are similar across endocrine disorders, although the methods used may vary, ranging from informal clinician communication to structured transition centers (155). Effective transition requires close collaboration between pediatric and adult endocrinologists, with shared information exchange and coordinated overlap in care to ensure continuity. Adolescents require developmentally appropriate healthcare that fosters knowledge, self-management skills, and increasing independence in managing their condition. Parents and caregivers should also remain actively involved during transition, allowing confidence to develop gradually as responsibility shifts to the young adult. Whether through standardized referral pathways, interdisciplinary collaboration, or dedicated transition services, efforts should focus on ensuring that adolescents with chronic endocrine disorders receive uninterrupted and age-appropriate care.

## 10 Conclusions and outlook

The level of clinical expertise applied during diagnosis and treatment plays a critical role in determining long-term outcomes and prognosis in patients with CP (156, 157). To promote uniform standards of care, the Pituitary Society has established criteria for centers of excellence specializing in the management of pituitary tumors (158). Support from professional societies and health authorities is essential to implement and maintain these standards. However, the establishment of such specialized centers requires substantial infrastructure, which may not be feasible within all healthcare systems. In such settings, alternative models, such as multicenter networks facilitating reference-based evaluations, should be considered to ensure high-quality care (159). In parallel with organizational improvements, ongoing research efforts aim to advance therapeutic strategies for CP, including the development of targeted treatments, with the ultimate goal of improving long-term patient outcomes (160).

Acquired hypothalamic obesity following CP represents a distinct clinical syndrome characterized by rapid and persistent weight gain. Effective therapeutic options remain a major unmet clinical need, as conventional lifestyle interventions and pharmacological treatments used for common obesity generally fail to address the specific pathophysiological mechanisms underlying acquired hypothalamic obesity after CP. Early therapeutic intervention is essential to reduce mortality and overall disease burden, including debilitating symptoms that negatively affect patient QoL, increase caregiver burden, and elevate the risk of cardiometabolic complications. Although acquired hypothalamic obesity is a complex neuroendocrine disorder involving multiple hypothalamic pathways, MC4R signaling has emerged as a key therapeutic target. Impairment of the α-melanocyte-stimulating hormone (α-MSH)/MC4R signaling pathway represents a major contributor to dysregulated energy homeostasis within the broader neuroendocrine and autonomic network, leading to manifestations such as hyperphagia and reduced energy expenditure. Weight reduction achieved through MC4R agonism is thought to result from restoration of energy balance via suppression of hunger signals and enhancement of REE.

Recent data of a randomized controlled phase 3 trial (55) provide, for the first time, encouraging evidence that the MC4R agonist setmelanotide may substantially improve the historically limited and often unsatisfactory management of patients with hypothalamic dysfunction associated with hypothalamic obesity. The availability of a safe and effective pharmacological intervention, addressing not only metabolic parameters but also key psychosocial features such as hyperphagia and QoL, might be a game changer to meaningfully improve the outcome (161). These developments underscore the need for the implementation of personalized treatment algorithms tailored to the heterogeneity and broad clinical presentation of hypothalamic obesity. Future patient cohorts with acquired hypothalamic obesity should benefit from standardized diagnostic approaches, systematic risk stratification, and prospective evaluation of multimodal therapeutic strategies. Such approaches should integrate optimized pituitary hormone replacement with combination pharmacotherapy, including agents targeting central pathways such as MC4R agonists (161). The adoption of these structured and individualized treatment paradigms may ultimately translate into significant improvements in clinical outcomes and QoL for this highly burdened and underserved patient population (162).
